# Oxygen Reductases in Alphaproteobacterial Genomes: Physiological Evolution From Low to High Oxygen Environments

**DOI:** 10.3389/fmicb.2019.00499

**Published:** 2019-03-18

**Authors:** Mauro Degli Esposti, Marek Mentel, William Martin, Filipa L. Sousa

**Affiliations:** ^1^Center for Genomic Sciences, UNAM Campus de Cuernavaca, Morelos, Mexico; ^2^Faculty of Natural Sciences, Department of Biochemistry, Comenius University in Bratislava, Bratislava, Slovakia; ^3^Institute of Molecular Evolution, University of Düsseldorf, Düsseldorf, Germany; ^4^Division of Archaea Biology and Ecogenomics, Department of Ecogenomics and Systems Biology, University of Vienna, Vienna, Austria

**Keywords:** oxygen, terminal oxidases, alphaproteobacteria, endosymbiosis, mitochondria, copper proteins, bacterial evolution

## Abstract

Oxygen reducing terminal oxidases differ with respect to their subunit composition, heme groups, operon structure, and affinity for O_2_. Six families of terminal oxidases are currently recognized, all of which occur in alphaproteobacterial genomes, two of which are also present in mitochondria. Many alphaproteobacteria encode several different terminal oxidases, likely reflecting ecological versatility with respect to oxygen levels. Terminal oxidase evolution likely started with the advent of O_2_ roughly 2.4 billion years ago and terminal oxidases diversified in the Proterozoic, during which oxygen levels remained low, around the Pasteur point (ca. 2 μM O_2_). Among the alphaproteobacterial genomes surveyed, those from members of the Rhodospirillaceae reveal the greatest diversity in oxygen reductases. Some harbor all six terminal oxidase types, in addition to many soluble enzymes typical of anaerobic fermentations in mitochondria and hydrogenosomes of eukaryotes. Recent data have it that O_2_ levels increased to current values (21% v/v or ca. 250 μM) only about 430 million years ago. Ecological adaptation brought forth different lineages of alphaproteobacteria and different lineages of eukaryotes that have undergone evolutionary specialization to high oxygen, low oxygen, and anaerobic habitats. Some have remained facultative anaerobes that are able to generate ATP with or without the help of oxygen and represent physiological links to the ancient proteobacterial lineage at the origin of mitochondria and eukaryotes. Our analysis reveals that the genomes of alphaproteobacteria appear to retain signatures of ancient transitions in aerobic metabolism, findings that are relevant to mitochondrial evolution in eukaryotes as well.

## Introduction

The alphaproteobacteria are a large and diverse group of prokaryotes, members of which can grow under chemotrophic, phototrophic, lithotrophic, organotrophic, autotrophic, heterotrophic, parasitic, aerobic, anaerobic, or diazotrophic conditions (Garrity et al., 2005; Baldani et al., 2014; de Souza et al., 2014; Pujalte et al., 2014). Some alphaproteobacteria, in particular members of the Rhodospirillaceae (Baldani et al., 2014; Degli Esposti et al., 2016; Degli Esposti and Martinez-Romero, 2017), can do all of the above, depending on environmental conditions, the basis of physiological versatility residing in the presence, expression and regulation of genes encoded in the strain’s genome. Alphaproteobacteria display a wide spectrum of physiological traits central to energy metabolism and ecological adaptation, accordingly. They also have a special place in microbial evolution, because they are the bacterial group that brought forth the common ancestor of mitochondria (John and Whatley, 1975; Yang et al., 1985; Williams et al., 2007; Atteia et al., 2009; Abhishek et al., 2011; Thiergart et al., 2012; Degli Esposti et al., 2016) and hydrogenosomes (Martin and Müller, 1998; Müller et al., 2012) at the origin of eukaryotes.

Although the fossil record of prokaryotes is scant, alphaproteobacteria can be estimated to have a minimum age of roughly 2 billion years (Ga) from molecular dating of eukaryotes, since mitochondria originated from this class of proteobacteria. Fossil data place a minimum age on eukaryotes of 1.45 Ga (Javaux et al., 2001; Javaux and Lepot, 2018). Current molecular estimates for eukaryote age are in the range of 1.7–1.9 Ga (Parfrey et al., 2011; Betts et al., 2018). Because mitochondria were present in the eukaryote common ancestor (Embley and Martin, 2006; van der Giezen, 2009; Judson, 2017), alphaproteobacteria are at least as old as eukaryotes themselves (Betts et al., 2018).

Alphaproteobacteria not only participated in the origin of eukaryotic organelles (Ku et al., 2015), they have been coexisting with eukaryotes in Earth’s diverse environments for nearly 2 billion years. What kinds of environments? Views about the nature of Earth’s habitats over Ga time scales tend to focus on oxygen, for understandable reasons, because the appearance of oxygen changed the chemistry of the planet. Current consensus has it that cyanobacteria started producing oxygen at least 2.4 billion years ago (the great oxidation event, or GOE), although the initial onset might have begun slightly earlier at 2.7 Ga (Anbar et al., 2007; Lyons et al., 2014; Fischer et al., 2016).

**FIGURE 1:**
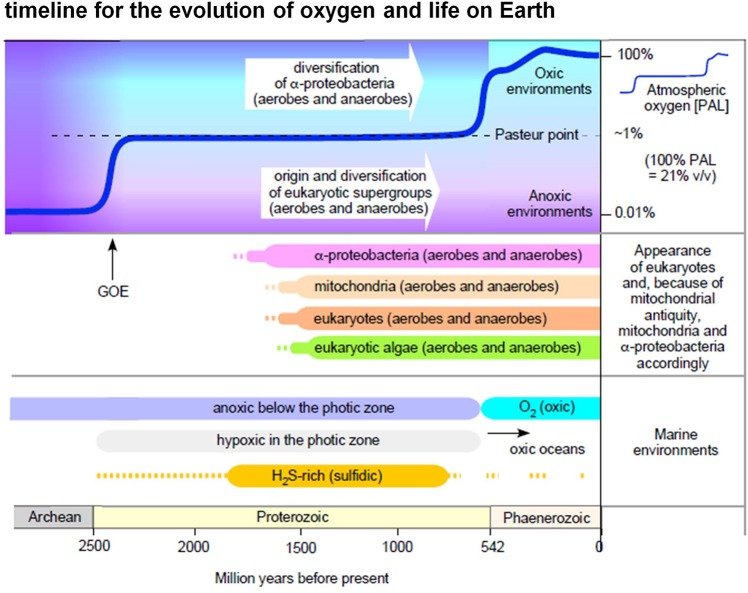
Oxygen in evolution. The figure summarizes selected major events in Earth history relating to the appearance of eukaryotic groups and the appearance of O_2_ in the atmosphere and in marine environments (see text, see also Mentel and Martin, 2008; Parfrey et al., 2011; Müller et al., 2012; Lyons et al., 2014; Fischer et al., 2016; Lenton et al., 2016). Note that in this diagram we do not interpret the light carbon signature at 2.4 Ga as a surge of high atmospheric O_2_ levels followed by an inexplicable decline to low levels (Lyons et al., 2014), rather we follow more traditional explanations of the light carbon surge involving methanogenesis (Hayes and Waldbauer, 2006).

Though O_2_ appeared in the geochemical record approximately 2.4 billion years ago, it took almost 2 billion years to accumulate to current levels in either the ocean or the atmosphere (Lyons et al., 2014; Fischer et al., 2016; Knoll et al., 2016; Javaux and Lepot, 2018). As a result, both eukaryotes and alphaproteobacteria existed in anoxic or low oxygen environments (Figure 1) for over a billion years before the critical rise of marine O_2_ at 580 million years ago, and the terminal rise of atmospheric O_2_ at 420 MY ago (Lenton et al., 2016). According to the ratio of Fe^3+^ to total Fe in hydrothermally altered basalts formed in ocean basins, Stolper and Keller (2018) estimated that deep-ocean oxygenation occurred as late as 541 million years ago and possibly as recently as 420 million years ago. Regardless of the exact timing of late deep-ocean oxygenation, oxygen had a substantial impact not only on the environment, but on the evolution of metabolism as well (Raymond and Segre, 2006; Sousa et al., 2016). It enabled the origin of O_2_-dependent biosynthetic pathways including cobalamin (Martens et al., 2002), chlorophyll (Sousa et al., 2013), and ubiquinone (Degli Esposti, 2017). The main contribution of O_2_ to metabolic pathways, however, was that it enabled the more complete oxidation of heterotrophic substrates than strictly anaerobic metabolisms or environments devoid of O_2_ and O_2_-derived high potential acceptors could support (Sousa et al., 2016).

**FIGURE 2:**
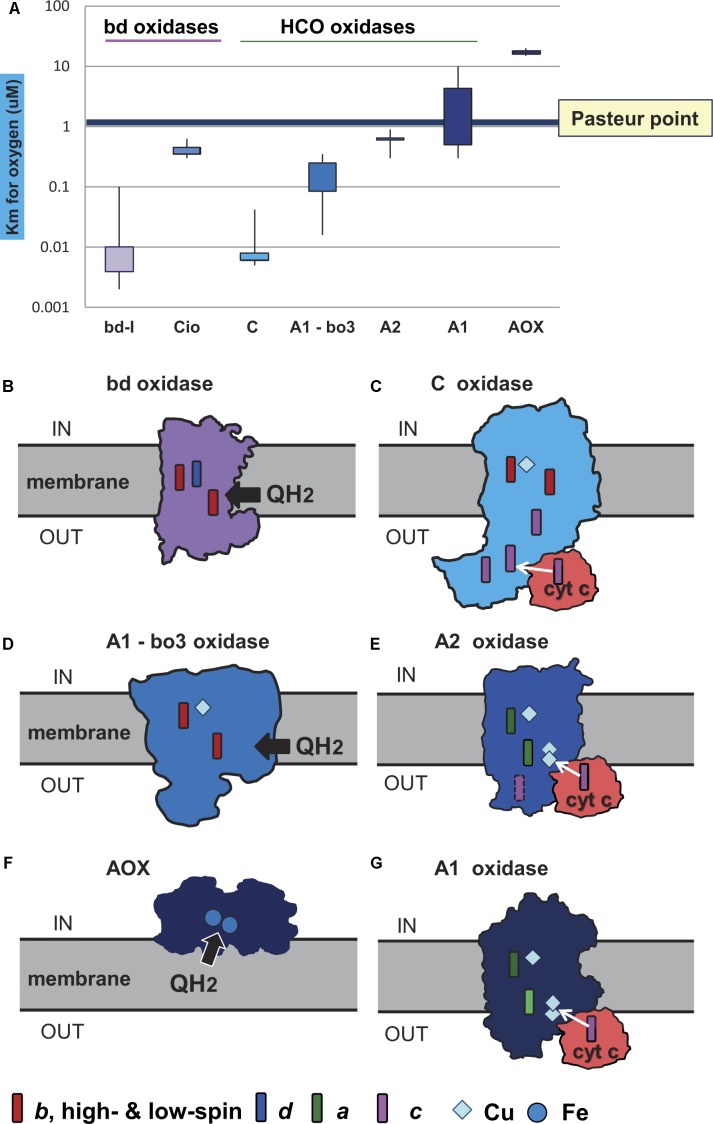
Oxygen affinity and structure of terminal oxidases. The structure of the membrane terminal oxidases is rendered as silhouette derived from 3D and biochemical data. Symbols for the heme and metal centers are shown at the bottom. **(A)** The illustration reports the quantitative values of reported *K*_m_ for oxygen in the indicated terminal oxidases. The values were taken from the primary literature (D’Mello et al., 1995, 1996; Jackson et al., 2007; Crichton et al., 2010; Krab et al., 2011; Miura et al., 2013; Ramel et al., 2013; Arai et al., 2014). Of note, the data for *bo*_3_ oxidase include *K*_m_ values reported for *E. coli* (D’Mello et al., 1996) and organisms other than alphaproteobacteria (Jackson et al., 2007). The *K*_m_ values for oxygen in the *bo*_3_ ubiquinol oxidases of alphaproteobacteria such as *Gluconobacter* are much higher than those in *E. coli*, from 3 to 7 μM (Miura et al., 2013; Richhardt et al., 2013); they are not considered in the graph for sake of clarity. **(B)** *Geobacillus thermodenitrificans* cytochrome *bd* ubiquinol oxidase (Safarian et al., 2016). **(C)** *Pseudomonas stutzeri cbb*_3_ oxidase (C family of HCO) from Buschmann et al. (2010). **(D)** *E. coli bo*_3_ ubiquinol oxidase of A1 type (Abramson et al., 2000). **(E)** *Thermus thermophilus caa*_3_ cytochrome *c* oxidase of A2 type (Lyons et al., 2012). **(F)** AOX (Shiba et al., 2013). **(G)** *aa*_3_ cytochrome *c* oxidase of *Paracoccus denitrificans* (Iwata et al., 1995), which is the reference bacterial protein for A1 type HCO (Pereira et al., 2001).

Prior to the advent of O_2_, all organisms on Earth were anaerobes and therefore harbored many O_2_ sensitive enzymes. Although scenarios have been proposed in which micro-aerophilic environments might have occurred on early Earth (Ducluzeau et al., 2014), trace amounts of O_2_ produced abiotically in the atmosphere would rapidly react with reductants and metals in Hadean oceans, such that trace abiogenic O_2_ would be hardly available for biological use. This is especially true if primary production prior to the advent of chlorophyll based photosynthesis was fuelled by geochemical H_2_, as available data suggests (Sleep et al., 2011; Arndt and Nisbet, 2012; Martin et al., 2018). The presence of O_2_ conferred selective advantage upon genes for enzymes that could detoxify O_2_, such as soluble diaphorases and alternative oxidases that do not conserve energy (Müller et al., 2012), and for enzymes such as superoxide dismutase, rubredoxin, and rubrerythrin (superoxide reductase) that could help microbes deal with reactive oxygen species. The initial function of terminal oxidases might not have been bioenergetic, but the removal of ambient or cytosolic levels of O_2_ as a toxin (Baughn and Malamy, 2004; Forte et al., 2016); although today, typical O_2_ removal systems are soluble NAD(P)H oxidases (diaphorases) (Müller et al., 2012) or ferredoxin dependent O_2_ reductases such as flavodiiron proteins (Di Matteo et al., 2008; Smutná et al., 2009), rather than terminal oxidases. The advent of O_2_ also impacted prokaryotic evolution by conferring selective advantage upon genes for terminal oxidases that could reduce O_2_ in the context of energy conservation. Membrane bound, quinone and cytochrome dependent oxygen reductases, generally called terminal oxidases, subsequently were selected in such a way as to allow microbes to cope with different levels of O_2_ while extracting energy from O_2_ reduction using respiratory chains (Baughn and Malamy, 2004; Han et al., 2011; Morris and Schmidt, 2013; Ducluzeau et al., 2014) (Figure 2).

Five points concerning oxygen in evolution should be underscored in regard to bioenergetic and physiological evolution among prokaryotes,: (i) life started out anaerobically, (ii) anaerobes invented O_2_ production, (iii) anaerobes invented enzymes for protective O_2_ detoxification, (iv) anaerobes invented enzymes for bioenergetic O_2_ utilization, and (v) anaerobes integrated O_2_ reduction into the broader scheme of bioenergetic evolution. During that process, anaerobes became facultative anaerobes (Martin and Sousa, 2015), obligate aerobes coming last in Earth history. In the general course of physiological evolution, anaerobiosis is the ancient form of respiration, facultative anaerobes followed, and strict aerobes are the latest of the latecomers.

**FIGURE 3:**
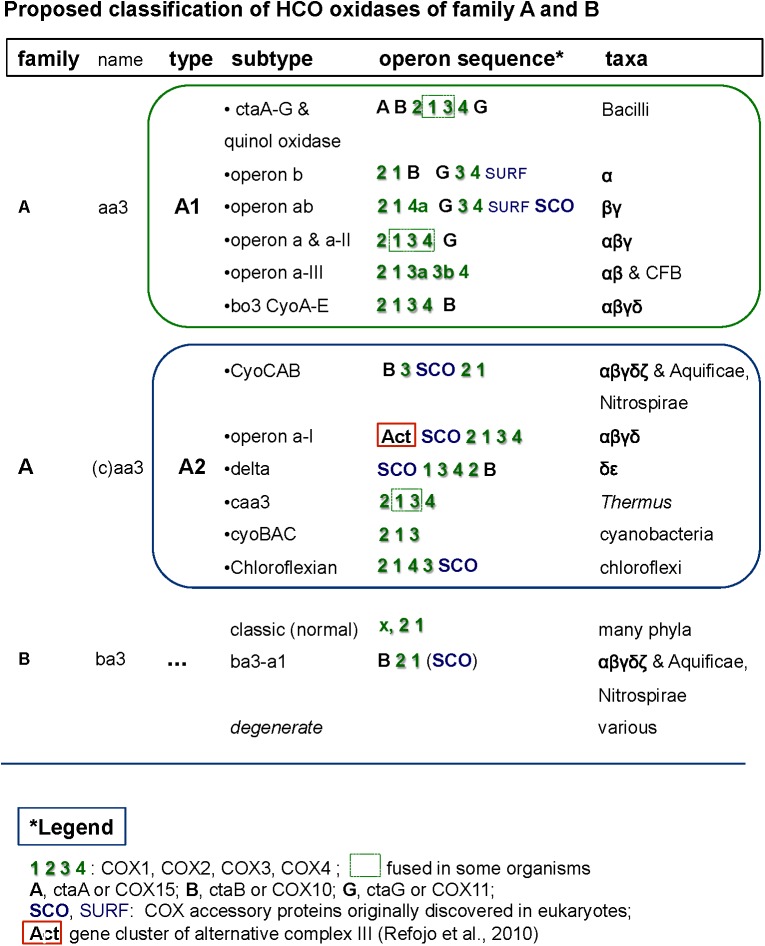
Comprehensive classification of family A and B terminal oxidases of the HCO superfamily including their operon organization. The figure shows examples of the different synteny organizations discussed in this paper. For comparative purposes, operons belonging to lineages outside of alphaproteobacteria are also shown.

Terminal oxidases, the enzymes responsible for the complete reduction of O_2_ to water, can use quinols, reduced cytochrome *c*, copper proteins, or even high-potential iron-sulfur proteins (Pereira et al., 2001) as electron donors for the reduction of oxygen (Figure 2). They belong to three different superfamilies, the largest of which includes protonmotive heme copper oxidases (HCOs) (Pereira et al., 2001; Sousa et al., 2012; Morris and Schmidt, 2013; Ducluzeau et al., 2014). HCO enzymes are characterized by having in their catalytic subunit (subunit I) a low-spin heme and a binuclear center composed of a high-spin heme, a copper ion (CuB) and a catalytic tyrosine residue which is covalently linked to one histidine ligand of CuB. Based on the conservation of the number and fingerprints of their proton channels (Iwata et al., 1995), HCOs are classified into three main families: A, B, and C (Pereira et al., 2001) (Figure 3). This classification is independent of the kind of hemes present in the catalytic subunit of the enzymes, and broadly corresponds with domain and operon organization, phylogenetic trees, and with the biochemical properties and oxygen affinities of the enzymes. The A family is further subdivided into A1 and A2 oxidases. Although in terms of proton translocation and oxygen affinity there may be no difference between these two subfamilies, their taxonomic distribution across prokaryotic lineages differs. Among eukaryotes, only the A1 family is present (Sousa et al., 2012). The superfamily of cytochrome *bd* oxidases contains only heme groups (Borisov et al., 2011) and is subdivided in two types, the *bd*-I typical of *E. coli* and the cyanide insensitive oxidase (Cio) typical of *Pseudomonas* (Cunningham et al., 1997), which have different evolutionary histories (Degli Esposti et al., 2015) and oxygen affinity (Figure 2A). The third superfamily is comprised of the alternative oxidases (AOX) of bacteria, plastids and mitochondria which oxidize quinols but do not contribute protonmotive force (Pennisi et al., 2016). Morris and Schmidt (2013) found that terminal oxidases other than AOX are present across many bacterial phyla.

Which group of prokaryotes first invented terminal oxidases? Common sense would have it that cyanobacteria, which are the source of all environmental O_2_, might have been the first group to evolve terminal oxidases because they were the first organisms on Earth to come into contact with O_2_. Indeed, many terminal oxidases occur in cyanobacteria, including A2, C and *bd*, with A2 type being the most common (Schmetterer, 2016). However, additional terminal oxidases that are widespread in proteobacteria and other phyla are lacking in cyanobacteria (Soo et al., 2017). In particular, cyanobacteria studied so far lack HCO terminal oxidases that belong to the A1 type and to family B (Pereira et al., 2001). Hence, these terminal oxidases likely evolved in other prokaryotic groups and then rapidly spread across different phyla via lateral gene transfer, LGT (Soo et al., 2017). Terminal oxidases together with complete bacterial respiratory chains have even migrated via LGT from the bacteria into the archaea (Nelson-Sathi et al., 2012; Wagner et al., 2017). All six types of terminal oxidases currently known are present in alphaproteobacteria (Figure 2).

In modern environments, the distribution of terminal oxidases among bacteria reflects lateral gene transfer and specialization to various ecological niches harboring different oxygen levels (Morris and Schmidt, 2013). In eukaryotes, the presence of terminal oxidases and enzymes of anaerobic energy metabolism also reflects ecological specialization, but due to differential loss, not lateral gene transfer (Müller et al., 2012; Martin, 2017). Here we investigate the number and nature of O_2_-reducing terminal oxidases in respiratory chains among sequenced and metagenomics-characterized alphaproteobacteria in order to survey energy metabolic diversity in the ancient lineage from which mitochondria arose.

## Results and Discussion

### Distribution, Expansion, and Loss of Terminal Oxidases in Alphaproteobacteria

While Morris and Schmidt (2013) searched genomes of predominantly cultured bacteria for the presence of terminal oxidases, here we focussed on alphaproteobacteria and metagenomic genome assemblies encoding >1000 proteins (Supplementary Figure S1 and Supplementary Table S1). Genome completeness was evaluated with different methods (Rinke et al., 2013; Simão et al., 2015) and taxa showing less than 90% coverage were subsequently excluded from further analysis (Supplementary Table S1 and Supplementary Figure S1). Ambiguous cases, for example when only one of the two catalytic subunits of cytochrome *bd* oxidase was present in genomes more than 90% complete, are indicated in light gray. The distribution of terminal oxidases among sequenced and metagenomic alphaproteobacterial genomes are given in Supplementary Table S1. Within our alphaproteobacterial dataset, we have found all six types of O_2_-reducing terminal oxidases. These complexes differ in their basic structure, subunit composition, redox groups, oxygen affinities and are widely distributed among microbes (Sousa et al., 2012; Degli Esposti, 2014; Ducluzeau et al., 2014; Marreiros et al., 2016). They are classified here on the basis of the widely recognized classification of HCO (Pereira et al., 2001; Sousa et al., 2012) and not on the kind of cytochromes (heme binding proteins) they contain. The heme classification, although important for historical reasons (Ferguson, 2001), often does not correlate with the functional proprieties of the enzymes. Moreover, it was shown that the QoxABCD from *Staphylococcus aureus* can assemble *in vivo* as a functional *aa_3_* or *bo_3_* enzyme according with the type of hemes produced in the cell (Hammer et al., 2016). Nitric oxide reductases (NORs) are evolutionarily related to the oxygen reductases and are considered as part of this superfamily (García-Horsman et al., 1994; Sousa et al., 2012; Ducluzeau et al., 2014) although performing a different reaction, the reduction of NO to H_2_O and N_2_O. In this work they were considered only in the initial survey (Supplementary Table S1). Our results indicate that the most common terminal oxidases within alphaproteobacteria belong to the A1 and C family of HCO, as previously noticed using complete genomes (Sousa et al., 2012). However, the new metagenomic diversity allowed us to extend the taxonomic distribution of the B type family to other alphaproteobacterial orders, such as Rhodobacterales and Rhodospirillales (Supplementary Table S1).

**FIGURE 4:**
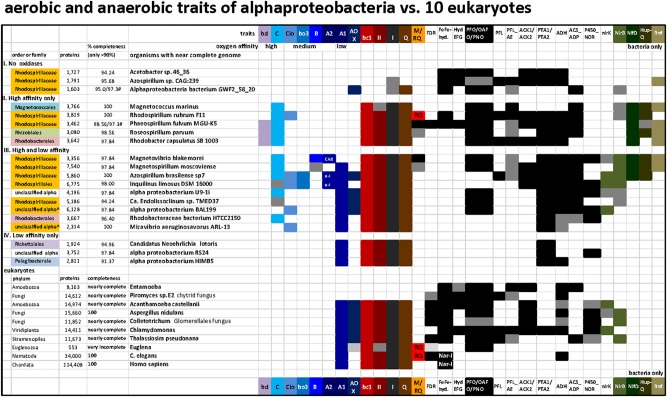
Distribution of terminal oxidases and various anaerobic traits shared between 20 bacteria and 10 eukaryotes in a selection of taxa representative of various lineages and metabolic combinations. The list of taxa includes representatives of the four categories of alphaproteobacteria that have been defined on the basis of the presence and oxygen affinity of terminal oxidases, as presented in the Section “Results and Discussion.” The taxa were selected from those listed in Supplementary Figure S1 to provide the broadest combinations of metabolic traits, which additionally included several traits of anaerobic metabolism that are shared with some eukaryotes (Atteia et al., 2013). The accession numbers for the key proteins that define each trait are listed in Supplementary Table S2; the distribution of ASCTI, malic enzyme, and fumarase (Müller et al., 2012) are not shown because they are widespread among the taxa considered (Supplementary Table S2) and to simplify the graph.

In a second step, the terminal oxidases were divided into low affinity and high affinity types on the basis of their different oxygen affinities (Figure 2). The high affinity oxidases include the bd-I type and C family oxidases, which have a *K*_m_ for O_2_ in the nanomolar range (D’Mello et al., 1996; Pitcher and Watmough, 2004; Morris and Schmidt, 2013; Arai et al., 2014). The low affinity oxidases include the Cio type of *bd* oxidases, HCO oxidases belonging to family A, and AOX oxidases (Figure 2). Although, to our knowledge, values of *K*_m_ for B family oxidases have not been reported so far, these enzymes are thought to have affinity for oxygen between that of family C and family A (Giuffrè et al., 1999; Radzi Noor and Soulimane, 2012) and were considered to be of intermediate oxygen affinity. Only AOX have *K*_m_ values for oxygen that are clearly higher than the Pasteur point (Figure 2 cf. Pennisi et al., 2016). A1 type oxidases including the mitochondrial cytochrome *c* oxidase (Krab et al., 2011) have *K*_m_ values for oxygen that are close to the Pasteur point, for example 4 μM (Arai et al., 2014), while A2 type oxidases have *K*_m_ values lower than the Pasteur point, for example 0.62 μM for *Desulfovibrio* (Ramel et al., 2013). A simple suggestion is that high affinity oxidases arose first in evolution in response to low O_2_ levels, that low affinity oxidases arose late in evolution when O_2_ had accumulated, and that B family oxidases could constitute an intermediate step in bacterial adaptation to increasing O_2_ levels after the GOE (de Vries and Schröder, 2002; Sharma and Wikström, 2014) (Figure 3, 4, and also Supplementary Table S1). As can be observed in Supplementary Table S1, the combination of oxidases differs significantly within the taxonomic family, and sometimes even within the same genus, reflecting the mosaic nature of aerobic prokaryotic chains.

On the basis of these simple operational criteria, genomes were sorted into four categories: (I) without oxidases; (II) containing only high affinity oxidases, *bd*-I quinol oxidase and C family *cbb*_3_ cytochrome *c* oxidase; (III) containing a mixture of high, intermediate and low affinity oxidases, and; (IV) containing only low affinity oxidases of the A1 family (with/without AOX) (Figure 4). An exception was made in the case of *Alphaproteobacteria bacterium* GWF2_58_20, whose genome encodes a putative AOX. However, all the remaining anaerobic traits present in this organism point to a strict anaerobic lifestyle and the presence of an AOX gene is most likely due to contamination. Thus, this organism was classified as strict anaerobe.

In Figure 4, representative alphaproteobacterial genomes were grouped by their tendency to reflect low oxygen (anaerobes), intermittent oxygen (facultative anaerobes) and high oxygen (low affinity oxidases) ecological strategies. The genus *Azospirillum* contains organisms whose metagenomic records indicate absence of all terminal oxidases and therefore can be categorized as strictly anaerobes, as shown at the top of Figure 4 (*Azospirillum* sp. CAG:239; see also Degli Esposti et al., 2016), but it also harbors diazotrophic organisms that possess low, high and intermediate affinity terminal oxidases such as *Azospirillum brasilense*, broadly categorized as facultatively anaerobes as shown in the middle of Figure 4. The collection of terminal oxidases in *Azospirillum* is thought to reflect pervasive contributions of LGT to their genomes (Wisniewski-Dyé et al., 2011), whereas the complete loss of terminal oxidases in other *Azospirillum* species and their relatives living in anaerobic niches presumably derives from ecological adaptation to anaerobiosis.

*Roseospirillum parvum* and *Rhodobacter capsulatus* appear in category II, but most likely have lost a low affinity A1 type oxidase since all the other members of their family have one or more of such oxidases (Supplementary Table S1). Accordingly, category II should include only taxa from the Rhodospirillaceae family plus some unclassified alphaproteobacteria. No member of the alphaproteobacteria sampled here has the high affinity *bd*-I as its sole terminal oxidase (Figure 4, Supplementary Figure S1 and Table S1). Conversely, *Magnetococcus*, the deepest branching alphaproteobacterium in some phylogenetic analyses (Esser et al., 2007; Degli Esposti et al., 2015; Ji et al., 2017), has a single C family oxidase of the *cbb_3_* type. Although *Magnetococcus marinus* shares magnetotaxis with Magnetospirilli, its anaerobic traits overlap with those of photosynthetic *R. rubrum* and *Phaeospirillum*, which do not have low or intermediate affinity oxidases and thus fall into category II (Figure 4, top, and Supplementary Figure S1). These members of the Rhodospirillaceae family can thus be considered among the ones containing ancient anaerobic traits of the class, from a physiological standpoint.

### Distribution of Anaerobic Traits Shared With Eukaryotes

For 100 selected lineages, we included enzymes of anaerobic energy metabolism germane to mitochondria and eukaryotes, in order to obtain a better picture of (facultative) anaerobic capacities within alphaproteobacteria (Figure 4). Additionally, we analyzed other bioenergetic systems that react with the same substrates of terminal oxidases, ubiquinol, and cytochrome *c*, plus the biosynthesis of membrane quinones (Degli Esposti, 2017). The presence of enzymes involved in anaerobic energy metabolism in eukaryotes is indicated in black (Figure 4 and cf. Supplementary Table S2). These enzymes are usually expressed in mitochondria, though sometimes in the cytosol (Müller et al., 2012) or in plastids in algal lineages (Atteia et al., 2013). The soluble enzymes of anaerobic fermentations have been retained in many different eukaryotic lineages and trace to the eukaryote common ancestor, they are also widespread among generalist alphaproteobacteria, underscoring the facultative anaerobic lifestyle of the eukaryote common ancestor and the role of its mitochondria in aerobic and anaerobic energy metabolism (Mentel and Martin, 2008; Müller et al., 2012). Among the surveyed genomes, none possess all the traits currently found in eukaryotic organisms, highlighting the continuous evolution and diversification of these lineages to different environments. In all surveyed genomes containing an A1 HCO, genes belonging to Complex I, II, and III, present in modern mitochondria were also found. In addition, many contained C type oxidase and genes predominantly associated with low oxygen or anaerobic lifestyles (see Supplementary Table S2 and Figure 4). As expected, within the group II organisms having only high affinity oxidases, a higher number of genes related with anaerobic traits was found, namely FeFe-hydrogenases and HydEFG complex which are mainly absent in organisms containing A1 type oxidase.

### Operon Organization of Alphaproteobacterial Terminal Oxidases

The genomic organization and its conservation can give clues regarding not only functional association of the genes but also regarding their evolutionary history. In the case of terminal oxidases, accessory proteins tend to be in genomic proximity to the catalytic subunits of the enzymes. However, due to genomic rearrangements, lack of gene synteny *per se* does not imply lack of functional association nor different evolutionary history along the broad time scales discussed in here. In the course of this work we encountered several operon subtypes of both A and B families of HCO that can be distinguished by the different sequence of the catalytic and accessory subunits. Some of these subtypes corresponded to previously introduced COX operon types (Degli Esposti, 2014) that for standardization of the nomenclature were here named according to the classification introduced by Pereira et al. (2001). Leaving aside the C family that appears to be more compact than other families (Sousa et al., 2012; Ducluzeau et al., 2014), family A includes two well defined subgroups, type A1 and type A2 (Pereira et al., 2001). According to their genomic organization, each of these types can be divided into several synteny subtypes, as shown in Figure 3. Therefore, and to express this feature, the variants of A family oxidases have a binomial nomenclature, in which the type is equivalent to a genus name and the subtype is equivalent to the species name of an organism (Figure 3). Following this binomial system, we have found in *Magnetovibrio blakemorei* an A2 type oxidase with a CyoCAB operon. The COX1 proteins of the previously known COX operon type a-I (the gene cluster typically associated with Act, cf. Refojo et al., 2010; Degli Esposti, 2014) are of A2 type, subtype a-I operon. Within our dataset, this operon was found in *Azospirillum brasilense* sp7 and *Inquilinus limosus*, two members of the Rhodospirillales order (Supplementary Table S2). The previously named COX operon type a and a-II (here A1 oxidases, subtype a) and the COX operon type a-III (the gene cluster with a characteristic doublet of COX3 homologs – Degli Esposti, 2014) named here as A1 type, subtype a-III were not so commonly found within the surveyed genomes. On the contrary, the mitochondria-like A1 oxidases containing the subtype b operon were found to be widely distributed among the group III and IV of the surveyed alphaproteobacterial genomes. Of note, Magnetospirilli have their own variant of the latter subtype that lacks the CtaB gene for accessory protoheme-farnesyl transferase, which is normally inserted between the gene for COX1 and that for CtaG (Degli Esposti, 2014) (see below). The conservation of operon structure is not restricted to alphaproteobacteria and variants of the A2 family have been previously found in deltaproteobacteria (subtype delta), cyanobacteria (subtype CyoBAC, cf. Soo et al., 2017) and Chloroflexi (subtype chloroflexian) to name a few. The diversity of operons found within this dataset argues in favor of a complex history and/or genomic reorganizations that occur within the genomes of alphaproteobacterial organisms throughout the evolution of this trait. Interestingly, the widespread presence of the A1 type b operon across organisms containing at least one A1 type oxidase contrasts with the scant distribution of A2 type oxidases among alphaproteobacteria (Figure 1 cf. Supplementary Table S1).

### *Magnetospira* and *Magnetovibrio* Look Like Living Fossils

**FIGURE 5:**
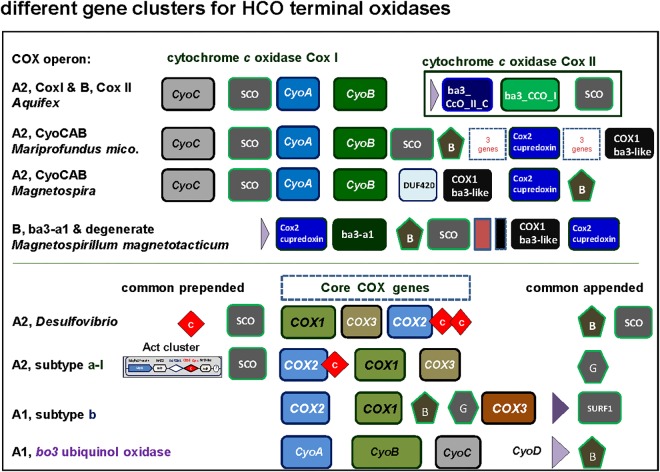
Graphical representation of gene clusters of A and B family terminal oxidases of the HCO superfamily. The gene clusters were graphically represented as before (Degli Esposti, 2014) with different color for each variants of catalytic subunit. Of note, among alphaproteobacteria the CyoCAB operon oxidase is present also in *Terasakiella* sp. PR1 (Supplementary Figure S1), a species previously classified among the Rhizobiales that has magnetotactic features, and metagenomic assembled genomes of other classes that remain poorly characterized. See Supplementary Figure S2 for phylogenetic trees.

*Magnetovibrio* and *Magnetospira* are marine Rhodospirillaceae distantly related to freshwater *Magnetospirillum* species. Recently, the polyphyletic nature of Rhodospirillaceae was shown by extended phylogenetic analysis (Parks et al., 2018). This family contains a variety of taxa with widely different physiology, as also indicated from our data (Figure 4). In the case of *Magnetovibrio* and *Magnetospira*, however, the physiological properties of aerobic metabolism are unique to both the family and the whole class of alphaproteobacteria – with the exception of another magnetotactic organism, *Terasakiella* sp. PR1 or *magnetica*, whose genome is less than 60% complete (Supplementary Table S1). Like all Magnetospirilli, these organisms thrive in low oxygen, around 1 μM O_2_, but have a more pronounced anaerobic metabolism than *Magnetospirillum* strains (Williams et al., 2012; Bazylinski et al., 2013; Ji et al., 2014; Li et al., 2014). Their genome contains the A2 type CyoCAB operon that is related to the A2 oxidase from *Aquifex* (Figure 5) (Prunetti et al., 2011) instead of the A1 type oxidase of other Magnetospirilli (Degli Esposti, 2014). This operon contains multiple genes coding for SCO proteins, for synthesis of cytochrome *c* oxidase (Schulze and Rödel, 1988), which bind and transport Cu and are normally involved in the assembly of the binuclear CuA present in subunit II of non-quinol oxidizing HCO enzymes from the A and B family (Banci et al., 2007; Sousa et al., 2012). SCO genes are also present in gene clusters fused with the Alternative Complex III (Act) which corresponds to the subtype a-I operon. The subtype a-I operon is distributed among alpha-, gamma- and also deltaproteobacteria (Refojo et al., 2010; Degli Esposti, 2014) and is usually associated with genes coding for A2 oxidases enzymes (Refojo et al., 2010) (Figure 3).

SCO proteins are found in the gene cluster of the A2 oxidase CyoCAB operon and in the operon of the putative B type (ba_3_-a1) oxidase present in *Magnetospirilli* (Figure 5). Interestingly, this and additional sequences which tend to form a sister clade of *bona-fide* B oxidases lack the catalytic tyrosine responsible for the full reduction of O_2_ to water. Although none has been biochemically characterized to date, according to the O_2_-reductases community (see for instance Ducluzeau et al., 2014) the function of the enzyme as O_2_ reductase is questioned and indications (albeit indirect) regarding their nitric oxide activity exist, such as the increase in transcription levels under conditions that favor nitric oxide production (Cho et al., 2006). Thus, we opt to consider this enzyme as a *degenerate* B family oxidase, a broad term encompassing diverse forms of oxidases that may have lost the oxygen reductase function of HCO or specialized different one, and whose *in vivo* function awaits biochemical characterization. In the other alphaproteobacteria here surveyed, the SCO proteins are not in gene clusters for either A and B family oxidases, but located elsewhere in the genome, as seen in the cases of *P. denitrificans* and *Rhodobacter sphaeroides* genomes (Dash et al., 2015). Conversely, SCO proteins are often present in B and A2 type operons (Figure 3, 5) of alphaproteobacteria possessing various anaerobic traits (Figure 4). Cu is absolutely required for the assembly of HCO (Ducluzeau et al., 2014; Bhagi-Damodaran et al., 2017) and has low bioavailability in oceans (Anbar, 2008). Indeed, bacteria that thrive under euxinic conditions, for example sulfate-reducing *Desulfovibrio*, encode SCO proteins in the operon of their A family oxidases (Figure 3, 5; see also Ramel et al., 2015). It is therefore possible that the A2 oxidase containing the rare CyoCAB operon enriched in SCO genes of *Magnetovibrio* and *Magnetospira* constitutes a relic of ancient euxinic conditions in proterozoic oceans, which has survived in their genome. Of note, SCO genes were also previously found in some beta- and gammaproteobacterial A1 type oxidases with subtype b operons (Banci et al., 2007; Degli Esposti, 2014).

### Cu Assembly Proteins and the Acquisition of Low Affinity Terminal Oxidases

Copper holds clues about oxygen history. The reason is that Cu has extremely low bioavailability (ca. 10^-17^ M) in the presence of sulfide (Williams and Fraústo Da Silva, 2003; Decker and van Holde, 2011), as has been common in anoxic conditions for as long as sulfate reducers have existed. Strict anaerobes are generally devoid of Cu-containing proteins (Ridge et al., 2008). An adage among physiologists has it that “copper is late,” meaning that the presence of Cu in a protein indicates an origin subsequent to the origin of O_2_. Although Proterozoic deep ocean chemistry following the GOE is thought to have been dominated by anoxic conditions (Reinhard et al., 2013), coastal surface waters might have had oxygen gradient zones (Poulton et al., 2010; Bazylinski et al., 2013). Except for NOR, all HCOs require Cu. Copper is also present in the catalytic site in methane monooxygenase (an O_2_-dependent enzyme) and in enzymes of the denitrification pathway (Ridge et al., 2008). Assembly proteins distantly related to the transmembrane type of ctaG which assists HCO oxidases are required for Cu assembly in these proteins (Lawton et al., 2016).

**FIGURE 6:**
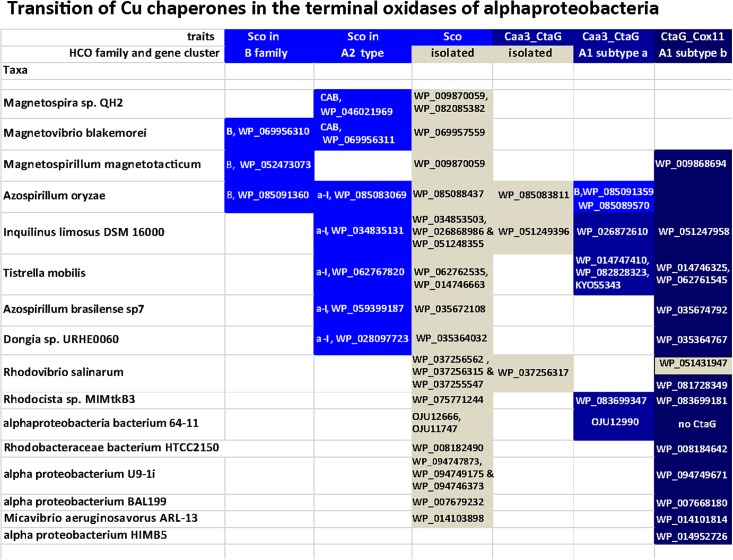
Distribution of Cu assembly proteins of different families along various alphaproteobacteria. Taxa were selected from Supplementary Figure S1 and Figure 4 to represent all major variations in the combination of Cu-binding assembly proteins that are present in alphaproteobacteria, either in isolated gene clusters or associated with diverse operon subtypes of HCO oxidases (see text).

The insertion of the Cu atom in the oxygen-reducing binuclear center requires the action of Cu assembly proteins (chaperones) that belong to two different families: the **Caa3_CtaG** transmembrane proteins (PFAM family PF09678) first characterized in *Bacillus* (Bengtsson et al., 2004) and the **CtaG_Cox11** family of periplasmic Cu assembly proteins (PFAM family PF04442), which are close homologs of eukaryotic Cox11 (Banci et al., 2004). Different Cu assembly proteins can be present either in isolated gene clusters or within different operon subtypes of A oxidases, similarly to SCO proteins (Figure 6). CtaG proteins that are in isolated gene clusters tend to branch deep in phylogenetic trees (M.D.E., unpublished data). Members of the Caa3_CtaG family are distantly related to *Deinococcus* proteins involved in the CopCD system used for eliminating Cu toxicity (Lawton et al., 2016) and are found in Rhodospirillaceae, Chloroflexi, Bacilli and various other bacteria. In alphaproteobacteria, Caa3_CtaG are often associated with A1 oxidases containing subtype a operons (Figure 5 and results not shown). In a phylogenetic analysis considering mainly alphaproteobacteria, it was observed that COX1 and COX3 subunits of A1 type subtype a tend to branch earlier than those of A1 type subtype b operon, suggesting that the former were present earlier during alphaproteobacteria evolution (Degli Esposti, 2014). However, it cannot be excluded that these operons are present within other taxonomic affiliations, whose inclusion in the tree might show a different topology.

Based on their phylogenetic distribution, CtaG_Cox11 proteins, which so far only have been found in proteobacteria and mitochondria, appear to have originated within the proteobacteria (Banci et al., 2004, 2007). The distribution of different Cu assembly proteins among selected organisms is shown in Figure 6. Although many SCO genes are not directly associated with terminal oxidase genes, there is a preference of genomic localization of SCO proteins with B and A2 family over A1 enzymes. In the same way, Caa3_CtaG and CtaG_Cox11 genes are preferentially in the proximity of A1 type genes. During evolution, SCO proteins could have undertaken the insertion of Cu in the binuclear center, a function conserved for C oxidases (Banci et al., 2007; Thompson et al., 2012) and A2 oxidases from deltaproteobacteria. The *bo_3_* ubiquinol A1 oxidases, present in many alphaproteobacteria other than Magnetospirilli (Figure 4), lack SCO proteins (Figure 5, cf. Degli Esposti, 2014) since its subunit II lost the residues necessary for Cu_A_ binding (Abramson et al., 2000; Pereira et al., 2001).

Our results suggest that the CtaG_Cox11 proteins in the dataset of the study are restricted to either the gene clusters of the A1 oxidases or scattered in the genomes of alphaproteobacterial having only A1 oxidases (Figure 6 and data not shown). This might support the proposal that, in the alphaproteobacterial class, A1 oxidases may have been present later than B and A2 type oxidases. However, it cannot be entirely excluded that the observed organization of A2 operons are the result of a recent acquisition, for instance from Deltaproteobacteria with whom these organisms share operon similarities, followed by genomic rearrangements. Conversely, the distribution of CtaG_Cox11 proteins indicates that they might have originated within the alphaproteobacterial class, confirming earlier reports (Banci et al., 2004, 2007). This evidence can also be correlated with the structure and lower affinity of CtaG_Cox11 vs. the other form of the CtaG protein, as well as to the evolution of oxygen levels on earth (Figure 1). The dimeric structure of the CtaG_Cox11 protein indicates that the Cu atom in each monomer is bound to two conserved Cys residues exposed to the periplasm (Banci et al., 2004). This implies that the activity of this protein most likely requires non euxinic conditions, since the excess of sulfhydryl groups in euxinic oceans would have out-competed CtaG_Cox11 proteins for the binding of Cu and its delivery to nascent cytochrome *c* oxidases. In contrast, sequence alignment of diverse Caa3_CtaG proteins shows the presence of multiple conserved His, Asp, and Met residues that could function as Cu ligands as in CopCD proteins (Lawton et al., 2016). This type of Cu ligation is tighter than that of the CtaG_Cox11 protein and would compete well for Cu binding and delivery under euxinic conditions. Given these biochemical features, we surmise that Caa3_CtaG proteins might have evolved earlier and under more anaerobic conditions than CtaG_Cox11 proteins. Consequently, the analysis of the differential distribution of the diverse Cu assembly proteins (Figure 6) can reveal clues on the evolutionary origin of low affinity terminal oxidases. Our finding that CtaG_Cox11 proteins in the dataset of the study are restricted to either the gene clusters of low affinity A1 type oxidases or scattered in the genomes of alphaproteobacteria having only A1 oxidases (Figure 6) indicate that these oxidases might have been present in alphaproteobacteria later than B and A2 type oxidases.

### Alphaproteobacterial HCOs: Can O_2_ Affinity Provide a Clue for Their Evolution?

There are two main views about terminal oxidase evolution among prokaryotes. One is that terminal oxidases were present in the last universal common ancestor (LUCA) and have been inherited vertically along microbial lineages during evolution, such that their distribution is the result of lineage divergence (Castresana and Saraste, 1995; Brochier-Armanet et al., 2009). The other is that life started off anaerobically and stayed anaerobic until the advent of oxygen-producing photosynthesis about 2.4 billion years ago (Fischer et al., 2016), whereby LGT subsequently distributed terminal oxidases among prokaryotic phyla, rendering their modern genomic distribution independent, to a large extent, of their evolutionary origin (Martin and Sousa, 2015; Soo et al., 2017; Weiss et al., 2018). LGT is a very real and pervasive process in the prokaryotic world (Wagner et al., 2017) and over long time spans it decouples physiology from phylogeny, both for photosynthesis (Brinkmann et al., 2018; Martin et al., 2018) and respiration (Figure 4).

The terminal oxidases of alphaproteobacteria differ in terms of oxygen affinity along a scale of *K*_m_ values ranging from nanomolar to micromolar (Figure 2A). For comparison, the O_2_ concentration in seawater at equilibrium with our present 21% v/v atmosphere at 25°C is on the order of 250 μM (Morris and Schmidt, 2013). Modern O_2_ levels arose late in evolution, however. During the time in which oxidases arose and diversified, roughly 2.4 billion years ago to *circa* 600 million years ago, atmospheric O_2_ was roughly 1% of present atmospheric levels (∼0.2% v/v) or less (Lyons et al., 2014; Javaux and Lepot, 2018; Stolper and Keller, 2018). Oxygen content corresponding to 1% PAL is very close to the Pasteur point (2.2 μM, 1% of present atmospheric level), the O_2_ concentration at which facultative anaerobes like *E. coli* start to respire O_2_ (Ducluzeau et al., 2014).

Here we have suggested that oxygen reductases of differing O_2_ affinity might have arisen in accordance with oxygen availability, with high affinity enzymes having emerged first and low affinity enzymes coming later in evolution. Because lateral gene transfer has substantial impact on terminal oxidase evolution (Soo et al., 2017), straightforward phylogenetic evidence to support our suggestion is generally problematic and ecophysiological factors bear heavily on terminal oxidase distribution. Han et al. (2011) proposed that adaptation from high O_2_ to low O_2_ environments could have been significantly impacted by bioenergetic factors. They pointed out that the four electron O_2_ reduction reaction is sufficiently exergonic at 1 nM O_2_ such that the low proton pumping stoichiometries typical of B- and C-type families is not a result of thermodynamic constraint, and furthermore that improved O_2_ diffusion to the active site of the B- and C-family enzymes might maintain physiologically relevant reaction rates at low O_2_ concentrations. Han et al. (2011) also stressed that the presence of different terminal oxidases in the genomes of many bacteria indicate physiological relevance, because bacteria typically do not maintain functionally related genes or operons without good physiological reason. Hence, our general reasoning is similar to that reported by Han et al. (2011), although the vector of O_2_ adaptation that we propose — from low to high O_2_ environments during geological time (Figure 1) — differs and the two interpretations, while both being physiologically founded, are by no means mutually exclusive.

## Conclusion

Both alphaproteobacteria and eukaryotes arose and diversified during a phase of Earth history in which oxygen levels were lower than today (Figure 1). Both lineages were highly diversified when oxygen made its rise to modern levels roughly 430 million years ago (Lenton et al., 2016; Stolper and Keller, 2018). Accordingly, most of alphaproteobacterial history and most of eukaryotic history can be summarized as “life at the Pasteur point,” which fits well with the distribution of aerobic and anaerobic eukaryotic lineages (Müller et al., 2012). It seems reasonable to propose that terminal oxidases of differing O_2_ affinity arose in a temporal sequence, from high affinity to low affinity following the gradual emergence of O_2_. LGT has distributed terminal oxidases from bacteria to archaea, yet the different Cu assembly proteins figure into evolutionary transitions in oxygen utilization within alphaproteobacteria. The distribution of terminal oxidases of differing oxygen affinity, often co-occurring with enzymes of anaerobic fermentations (Figure 4), reveals that biochemical traces of the anaerobic past may be still preserved in alphaproteobacterial genomes. A strictly aerobic metabolism similar to that typical of mammalian mitochondria is rare among alphaproteobacteria and is best understood as a result of ecological specialization to continuously aerobic habitats: life on land and above the soil line over the last 430 MY. In summary, different lineages of alphaproteobacteria and eukaryotes have undergone evolutionary specialization to high oxygen, low oxygen, and anaerobic habitats, and some have remained facultative anaerobes that are able to generate ATP with or without the help of oxygen. This physiology is still predominant in sequenced extant alphaproteobacteria and, by inference, also in their lineages from which mitochondria evolved.

## Materials and Methods

Sequence searches were undertaken with two independent complementary approaches that were then merged in a consensus picture for the presence or absence of aerobic and anaerobic traits in bacterial and eukaryotic genomes. The first approach consisted in genome-wide, computer-assisted analyses using reference proteins that are representative of metabolic traits, or each subunit of the various terminal oxidases as queries in Blast searches (Altschul et al., 1997). As a general rule, the protein searches had a threshold of 25% sequence identity and/or E-values below 1e^-10^ (Atteia et al., 2013; Marreiros et al., 2013; Martin and Sousa, 2015). HCOs were classified into the different families using the same approach as in Sousa et al., 2012).

The second approach was based upon searches in the NCBI protein and gene webpages using multiple keywords that had empirically shown to retrieve most of the homologs of a given protein in previous works (Degli Esposti, 2014, 2017; Degli Esposti et al., 2015). Protein homologs of each query were recognized by their conserved domain structure (CDD, Marchler-Bauer et al., 2015) and hydropathy profile using the server TMpred^1^ (Degli Esposti et al., 2015). In several cases, manually curated sequence alignments were additionally used to refine the assignment of candidate homologs (Degli Esposti, 2017). Searches were extended to comparable sets of all other classes of proteobacteria and all available genomes of Nitrospirae. The results of the two approaches were then combined and all cases of discrepancy carefully examined for potential sources of false negatives or positives, as well as human and computer errors. Remaining cases of potential ambiguity with respect to presence or absence of a given trait, for instance the presence of only one of the two catalytic subunits of *bd* oxidase in genomes more than 90% complete, were rendered in light gray in display material such as Figure 4.

A initial literature search identified around 200 genomic records to be analyzed. The initial set of alphaproteobacterial genomes was compiled from taxa that are predominantly uncultured, unclassified or derived from metagenomic assemblies, have around 1000 or more coded proteins, and could represent all known families and genera of the alpha class (Supplementary Table S1). This set was subsequently streamlined to 100 taxa (Supplementary Figure S1) with complete or high quality genomes having more than 90% genome coverage and at least 16 tRNA genes, as recently recommended (Bowers et al., 2017). Genome completeness was evaluated with different methods (Rinke et al., 2013; Simão et al., 2015) and taxa showing less than 90% coverage were excluded from analysis (Supplementary Table S1). The genomes of 10 eukaryotic taxa were additionally analyzed for the same traits (Figure 4) to expand and refine previous studies (Atteia et al., 2013).

The presence of terminal oxidases was color coded with increased intensity of blue following their decreased affinity for oxygen (Figure 1, 2, 4). Other bioenergetic traits were coded with various colors (cf. Degli Esposti and Martinez-Romero, 2017), while various anaerobic traits that are present in some eukaryotes and bacteria (Atteia et al., 2013) were rendered in black. The absence of a trait was always presented as a white box. See the legend of Figure 4 for further details on the definition of the examined traits.

Multiple sequence alignments were calculated using Clustal (Larkin et al., 2007) and maximum likelihood (ML) phylogeny reconstructed in IQ-tree (iqtree-omp version 1.5.5, Nguyen et al., 2015) with the best model (LG + I + G4) and 1000 bootstrap replicates. In addition, for selected sequences, further phylogenetic analysis was conducted as previously described (Degli Esposti et al., 2016; Degli Esposti, 2017) by combining preliminary Neighbour Joining (NJ) trees produced by wide Blast searches of HCO protein subunits with subsequent phylogenetic reconstruction using the program MEGA5 with selections of proteins that closely matched the topology of the preliminary trees. Such reconstructions were carried out with either the NJ or the ML approach using the standard Dayhoff scale of amino acid substitution and a minimum of 400 bootstrap replicates.

## Author Contributions

MDE and WM contributed conception of the study. MDE, WM, MM, and FS designed the study. MDE and FS performed bioinformatics analysis. All the authors contributed to the writing and revision of the manuscript, and read and approved the submitted version.

## Conflict of Interest Statement

The authors declare that the research was conducted in the absence of any commercial or financial relationships that could be construed as a potential conflict of interest.

## Footnotes

1 https://embnet.vital-it.ch/software/TMPRED_form.html
