# Supplementary material for: Oxygen Reductases in Alphaproteobacterial Genomes: Physiological Evolution From Low to High Oxygen Environments
Source: Front Microbiol. 2019 Mar 18;10:499. doi: 10.3389/fmicb.2019.00499 (PMC6431628; doi:10.3389/fmicb.2019.00499)
Supplement: Supplementary file 3 [file Image_1.pdf]

Supplementary material. It contains two Tables and two figures.

Table S1. List of ca. 200 alphaproteobacterial taxa and their aerobic functional traits as the basis for the distribution analysis of Supplementary Fig. S1 and Figs. 4 and 5 of the main text.

Table S2. Compilation of the accession numbers for all the key proteins of the metabolic traits listed in Fig. 4.

The table lists all the accession numbers (sometimes more than one per protein) for the proteins, either subunits or single protein enzymes, presented in Fig. 4. Besides the terminal oxidases and metabolic traits presented in Supplementary Fig. S1 with the same colour code, additional anaerobic traits were considered as they are shared by some alphaproteobacteria and eukaryotes (cf. Atteia et al., 2013). The acronyms for these anaerobic traits are as folows: FDR, fumarate reductase, membrane bound and reacting with Q or RQ; FeFe-Hyd., [FeFe]-hydrogenase HydA; HydEFG, maturases for [FeFe]-hyrogenase; PFO/OAFO/PNO, pyruvate:ferredoxin (flavodoxin) oxidoreductase PFO, 2-oxoacid:acceptor oxidoreductase (OAFO) or the eukaryotic variant PNO; PFL, pyruvate formate lyase; PFL_AE, pyruvate formate lyase activating enzyme (sets the glycyl radical); ACK1/ACK2, acetate kinase; PTA1/PTA2, phosphate acetyltransferase isoform 1 and 2; ADH, phosphotransacetylase bifunctional alcohol/ aldehyde dehydrogenase; ACS_ADP, acetyl-CoA synthase ADP forming; ASCTI, Acetate succinate CoA transferase (any of three types); malic enzyme; fumarase, fumarate lyase; P450_NOR, P450-related nitrate reductase of fungi; NirK, soluble nitrate reductase also present in some mitochondria. ASCTI, malic enzyme and fumarase are not shown in Fig. 4, also to simplify its graphical layout.

Fig. S1. List of 100 alphaproteobacterial taxa and their terminal oxidases as well other functional traits. 100 taxa were selected from those initially analysed and reported in Supplementary Table S1 so as to represent all major families of alphaproteobacteria and various unclassified taxa that had genome estimated to have at least 90% completeness, coding for more than 1000 proteins and at least 16 tRNA - therefore to be considered nearly complete (Bowers et al., 2017). The taxa were organized according to the presence and oxygen affinity of terminal oxidases in their genomes, as discussed in the text. The terminal oxidases are colour coded as in Fig. 2 while other metabolic traits are colour coded as reported previously (Degli Esposti and Martinez-Romero, 2017). Taxa labelled in orange on the left column of taxonomy were found to cluster within the family of Rhodospirillaceae by multiple phylogenetic trees (cf. Degli Esposti, 2017).

Fig. S2. Phylogenetic analysis of various COX1 proteins of A and B family terminal oxidases of the HCO superfamily. A) Maximum-likelihood phylogeny of the catalytic subunit I of selected HCOs. Alignment was performed in Clustal and the phylogenetic reconstruction in IQ-Tree (LG+I+G4, 1000 bootstraps). The tree is rooted between A and B family. B) **Representative NJ tree of COX1 proteins of A and B family terminal oxidases.** Representative COX1 proteins indicated by the accession number were first aligned by ClustalW and then the alignment was manually refined taking into account also the 3D structure of bacterial COX1 (Iwata et al., 1995). The phylogenetic analysis of the resulting alignment was undertaken following the ML approach with 500 bootstraps using the program MEGA5 as described previously (Degli Esposti, 2017).

Fig. S1.

	sheet 1, whole set		% completeness	master dataset for Supplementary Figure 1	bd	C	Cio	bo3	B	A2	A1	AOX	bc1	II	I	Q	M/RQ	FeFe-	PFO/(	NirB	NifD	Hup-(	Rnf
category		21-Feb-18		oxygen affinity		high		medium			low												
	order	proteins	at least 90%	organisms with near complete genome																			
I. No oxidases				anaerobes																			
	Rhodospirillales	1,791	95.68	Azospirillum sp. CAG:239																			
	Rhodospirillales	1,762	94.96	Azospirillum sp. CAG:260																			
	Rhodospirillales	1,727	94.24	Acetobacter sp. 46_36																			
	Rhodospirillales	1,775	89.2/89.9#	Acetobacter sp. CAG:977																			
	Rhodospirillales	1,603	95.0/97.3#	Alphaproteobacteria bacterium GWF2_58_20																			
II. High affinity only				facultatively anaerobic with high affinity terminal oxidases																			
	Magnetococcales	3,766	100	Magnetococcus marinus																			
	Rhodospirillales	3,819	100	Rhodospirillum rubrum F11													RQ						
	Rhodospirillales	3,462	98.56/97.3#	Phaeospirillum fulvum MGU-K5																			
	Rhodospirillales	3,703	97.84	Rhodospira trueperi																			
	Rhizobiales	3,080	98.56	Roseospirillum parvum																			
	Rhodobacterales	3,642	97.84	Rhodobacter capsulatus SB 1003																			
	Sphingomonadales	3,779	98.56	Zymomonas mobilis subsp. mobilis NCIMB 11163																			
	unclassified alpha	2,107	93.53	Thermopetrobacter sp. TC1																			
III. High and low affinity			100	facultatively anaerobes with multiple terminal oxidases																			
	Rhodospirillales	3,356	97.84	Magnetovibrio blakemorei						CAB													
	Rhodospirillales	3,693	100	Magnetospira sp. QH2						CAB													
	Rhodospirillales	7,540	97.84	Magnetospirillum moscoviense																			
	Rhizobiales	6,970	98.56	Terasakiella sp. PR1						CAB													
	Rhodospirillales	4,754	100	Caenispirillum salinarum AK4																			
	Rhodospirillales	5,860	100	Azospirillum brasilense sp7																			
	Rhodospirillales	6,775	100/98#	Inquilinus limosus DSM 16000																			
	Rhodospirillales	7,973	97.12	Rhodospirillales bacterium URHD0017																			
	Rhodospirillales	5,253	94.24	Rhodospirillales bacterium 69-11																			
	Rhodospirillales	2,588	92.09	Rhodospirillales bacterium RIFCSPLOWO2_12_FULL_58_28																			
	Rhizobiales	7,042	100 (97.8)	Rhodoplanes sp. Z2-YC6860													RQ						
	Rhodospirillales	5,250	96.40	Reyranella massiliensis																			
	Rhodospirillales	4,139	100	Thalassospira xiamenensis M-5																			
	unclassified alpha^	5,465	96.40	alpha proteobacterium Q-1																			
	Rhodospirillales	4,448	97.12	Rhodospirillales bacterium 70-18																			
	unclassified alpha	4,576	97.84	alpha proteobacterium AAP38																			
	Rhodospirillales	5,570	98.56	Acetobacter sp. SLV-7																			
	Rhodospirillales	6,061	97.12	Rhodospirillales bacterium URHD0088																			
	Rhodospirillales	5,289	97.84	Acetobacteraceae bacterium AT-5844																			
	unclassified alpha^	1,983	96.40	Alphaproteobacteria bacterium CG1_02_46_17																			
	Sphingomonadales	2,870	98.56	Erythrobacteraceae bacterium HL-111																			
	Sphingomonadales	3,106	94.24	Sphingomonadales bacterium 63-6																			
	Sphingomonadales	9,901	97.84	Sphingomonas sp. Root50																			
	Sphingomonadales	3,909	97.12	Sphingomonadales bacterium RIFCSPHIGHO2_01_FULL_65_20																			
	Rhizobiales	4,622	97.84	Afipia sp. NBIMC_P1-C1																			
	Rhizobiales	4,749	97.84	Afipia sp. OHSU_II-C2																			
	Rhizobiales	4,486	97.12	Rhizobiales bacterium 62-47																			
	Rhizobiales	7,786	96.40	Bradyrhizobium sp. Ai1a-2																			
	Rhizobiales	7,728	97.12	Bradyrhizobium sp. NAS96.2																			
	Rhizobiales	5,375	97.84	Rhodopseudomonas sp. AAP120																			
	Rhizobiales	4,236	97.12	Bradyrhizobiaceae bacterium SG-6C																			
	Rhizobiales	10,155	97.12	Bosea sp. PAMC 26642																			
	Rhizobiales	7,750	96.40	Bosea sp. Leaf344																			
	Rhizobiales	8,437	97.84	Aureimonas sp. Leaf454																			
	Rhizobiales	4,677	100 (96.4)	Aureimonas sp. AU40																			
	Rhizobiales	4,052	97.84	Devosia sp. 67-54																			
	Rhizobiales	4,296	100 (97.8)	Devosia sp. H5989																			
	Rhizobiales	3,687	97.84	Devosia sp. YR412																			
	Rhizobiales	3,726	97.12	Hyphomicrobium sp. CS1GBMeth3																			
	Rhizobiales	5,976	97.84	Beijerinckia sp. 28-YEA-48																			
	Rhizobiales	4,632	97.12	Microvirga sp. BSC39																			
	Rhizobiales	4,199	95.68	Methylocystis sp. LW5																			
	Rhizobiales	4,529	97.84	Methylosinus sp. PW1																			
	Rhizobiales	3,958	96.40	Pleomorphomonas koreensis																			
	Rhizobiales	7,239	97.12	Rhodovulum sp. ES.010																			
	Rhizobiales	4,804	96.40	Cohaesibacter marisflavi																			
	Rhizobiales	7,427	96.40	Methyloligella halotolerans																			
	Rhizobiales	5,582	97.84	Mesorhizobium sp. AA23																			
	Rhizobiales	3,285	94.96	Pseudochrobactrum sp. AO18b																			
	Rhizobiales	4,706	96.40	Ochrobactrum sp. P6BS-III																			
	Rhizobiales	4,460	97.84	Phyllobacterium sp. CL33Tsu																			
	Rhizobiales	6,494	100 (95.7)	Methyloceanibacter caenitepidi																			
	Rhizobiales	3,742	90.65	Rhizobiales bacterium 63-22																			
	Rhizobiales	5,228	90.65	Rhizobiales bacterium 62-17																			
	Rhizobiales	4,266	96.40	Rhizobiales bacterium 64-17																			
	Rhizobiales	9,156	97.84	Rhizobiales bacterium GAS113																			
	Rhizobiales	4,323	97.84	Rhizobiales bacterium NRL2																			
	Rhizobiales	4,400	97.84	Mongoliimonas terrestris (ex Rhizobiales MIMtkB18)																			
	Rhodobacterales	4,632	97.12	Rhodobacteraceae bacterium PD-2																			
	Rhodobacterales	4,470	95.68	Rhodobacterales bacterium 65-51																			
	Rhodobacterales	3,304	97.12	Rhodobacteraceae bacterium HLUCCO07																			
	Caulobacterales	2,831	95.68	Caulobacterales bacterium RIFCSPHIGHO2_01_FULL_70_19																			
	unclassified alpha	4,802	97.12	Alphaproteobacteria bacterium AO1-B																			
	Rhodobacterales	4,632	97.12	Rhodobacterales bacterium RIFCSPHIGHO2_02_FULL_62_130																			
	unclassified alpha	3,647	96.40	alpha proteobacterium Mf 1.05b.01																			
	unclassified alpha	4,196	97.84	alpha proteobacterium U9-1i																			
	Rhodospirillales	5,186	94.24	Candidatus Endolissoclinum sp. TMED37																			
	Holosporales	2,990	96.40	endosymbiont of Acanthamoeba sp. UWC8																			
	Rickettsiales	3,419	97.84	Rickettsiales bacterium Ac37b																			
III-3. Cio & low affinity				facultatively anaerobes with secondary reduction of terminal oxidases																			
	unclassified alpha	1,322	95.68	Alphaproteobacteria bacterium RIFCSPHIGHO2_02_FULL_40_34																			
	unclassified alpha	1,964	93.53	Alphaproteobacteria bacterium RIFCSPHIGHO2_02_FULL_46_13																			
	Rhizobiales	6,300	97.12	Methylobacterium sp. 190mf																			
	Rhizobiales	8,956	97.84	Rhizobiales bacterium GAS191																			
	Rhizobiales	4,437	97.12	Variibacter gotjawalensis																			
	Rhizobiales	5,116	97.84	Bosea sp. UNC402CLCol																			
	unclassified alpha^	1,422	94.24	Alphaproteobacteria bacterium 43-37																			
	unclassified alpha	1,643	93.53	Alphaproteobacteria bacterium 33-17																			
	unclassified alpha^	2,314	100	Micavibrio aeruginosavorus ARL-13																			
	unclassified alpha^	6,128	97.84	alpha proteobacterium BAL199																			
	unclassified alpha^	2,526	91.37	Alphaproteobacteria bacterium 41-28																			
	Rhizobiales	1,685	96.40	Candidatus Tokpelaia hoelldoblerii																			
	Rhodospirillales	5,596	100	Dongia sp. URHE0060																			
IV. Low affinity only				strictly aerobes																			
	Rickettsiales	1,924	94.96	Candidatus Neoehrlichia lotoris																			
	unclassified alpha	3,752	97.84	alpha proteobacterium RS24																			
	Rhodobacterales	6,923	97.84	Rhodobacteraceae bacterium SB2																			
	Pelagibacterales	2,811	91.37	alpha proteobacterium HIMB5																			
	unclassified alpha	1,549	92.09	Alphaproteobacteria bacterium RIFCSPHIGHO2_12_42_13																			
				eukaryotes													RQ						

Fig. S2

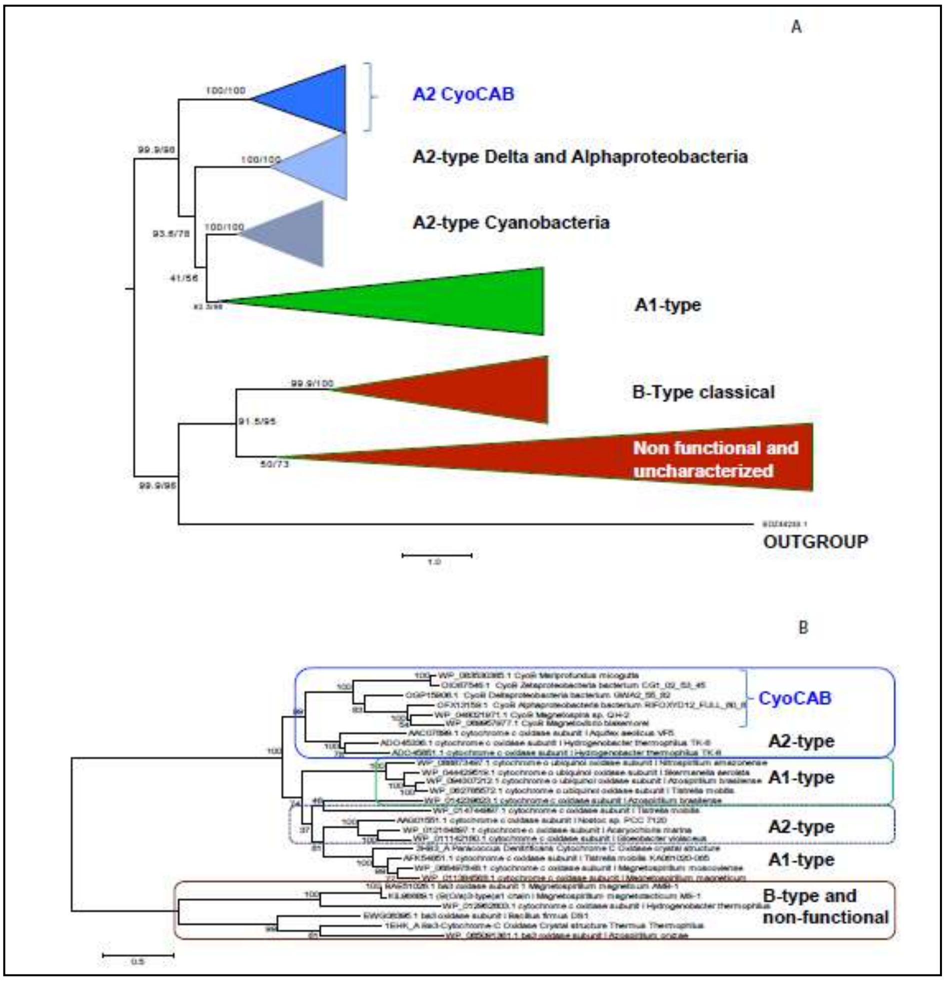
A
A2 CyoCAB
A2-type Delta and Alphaproteobacteria
A2-type Cyanobacteria
A1-type
B-Type classical
Non functional and uncharacterized
OUTGROUP
B
CyoCAB
A2-type
A1-type
A2-type
A1-type
B-type and non-functional
